# Dog owners’ job stress crosses over to their pet dogs via work-related rumination

**DOI:** 10.1038/s41598-025-01131-x

**Published:** 2025-05-15

**Authors:** Tanya Mitropoulos, Allison Andrukonis

**Affiliations:** 1 Department of Psychology, Virginia Tech109 Williams Hall, 890 Drillfield Dr, Blacksburg, VA 24061 USA; 2 School of Animal Sciences, Virginia Tech, 3110 Litton Reaves Hall, 175 West Campus Dr, Blacksburg, VA 24061 USA; 3 Present Address: Department of Psychology, Radford University, 5101 Hemphill Hall, 965 E Main St, Radford, VA 24141 USA; 4 Present Address: Department of Animal and Dairy Sciences, University of Wisconsin-Madison, 287 Animal Sciences Building, 1675 Observatory Dr, Madison, WI 53706 USA

**Keywords:** Crossover, Emotional contagion, Pet welfare, Rumination, Job stress, Animal behaviour, Stress and resilience, Psychology

## Abstract

Job stress can have well-being consequences for the employee’s loved ones through crossover: the transference of work-related stress from an employee to their family members. Pet dogs—who most Americans view as family members—may also be susceptible to crossover. Given prior support for dogs’ abilities to perceive and absorb a human’s emotions via emotional contagion, we expected that pet dogs of owners with higher job stress would themselves be more stressed. We anticipated that work-related rumination, or the tendency to continue thinking about work during leisure time, would explain this relationship. All variables were measured using self-report scales administered in an online survey, and dogs’ stress was captured in two ways: as owner-perceived and behaviorally indicated. The sample included 85 employees and their pet dogs. Controlling for home stress, we found that job stress related to behaviorally indicated stress in dogs. Work-related rumination explained this relationship. This study unites organizational research with companion animal welfare and supports the presence of crossover in the dog-owner bond. Based on these findings, employed dog owners might benefit from avoiding work-related rumination when at home to protect the well-being of man’s best friend.

**Supplementary Information:**

The online version contains supplementary material available at 10.1038/s41598-025-01131-x.

## Introduction

Job stress is considered by many to be an epidemic in the United States^1^, affecting 77% of American workers^2^. Organizational psychology has demonstrated that stress arising from an employee’s job can have detrimental effects not just for the employee, but for their loved ones too. Most often studied between spouses, wives have been found to “catch” the work-related stress of their husbands^3–5^. Children of parents with more job stress had more negative interactions with their parents^6,7^. This contagion of work-related stress between loved ones is called crossover^8^. While this phenomenon is well studied from an employee to the loved people of their households, other beloved yet under-studied household members exist: pets.

Over 97% of American pet owners consider their pet to be a member of the family^9^. Pet dogs may be particularly vulnerable to a crossover effect due to dogs’ uniquely advanced socio-cognitive abilities in relation to human communication^10,11^. Dogs are highly sensitive animals who experience emotional contagion and “catch” the feelings of other individuals, including humans^12^. Dogs have been shown to experience increases in stress both behaviorally and physiologically when their owner experiences a rise in stress^13,14^ or when hearing a human cry^12,15^. On a chronic level, pet dogs have displayed signs of synchronization with their owners’ stress levels, showing stress coping capabilities that relate to their owner’s personality (as measured by salivary cortisol variability^16^) and alignment between their own stress level and their owner’s (as measured by hair cortisol concentration^17^).

To our knowledge, a link between an owner’s job stress and their pet’s stress has not been studied. Yet, the mechanisms by which an employee’s job stress crosses over to their human family members^8^ could apply within human-dog dyads too. One pathway is direct transmission, wherein an individual “feels” the stress of their partner in a mechanism resembling empathy. This mechanism mirrors the phenomenon of emotional contagion previously observed in dogs, but applied specifically in the context of job stress. Given that dogs have been shown to experience increases in stress when their owner experiences a rise in stress^13^, even in the context of long-term stress^17^, dogs may similarly be more stressed if owned by a person with more job stress.

Another pathway for the crossover of job stress is indirect transmission^8^, wherein a person’s experience of job stress may result in behavioral changes that influence the dog-owner relationship. For example, a job-stressed owner may interact less with their dog, as stressed employees have been shown to do with their spouse^18^. Job stress may arise from long work hours^19^, which could lead an owner to spend less time caring for their pet, such as taking shorter walks or forgetting to feed their pet. With less care, a pet is likely to experience a rise in stress. An owner facing more job stress may also be more likely to use more aggressive methods when disciplining their dog, mirroring the positive association found between parental stress and aggressive disciplining of children^20^. This side effect of an owner’s job stress could lead to a dog who is more stressed.

In addition to job stress, work-related rumination has been shown to associate with poor familial relationships. Work-related rumination is the engagement in perseverative thoughts about work during non-work time^21^. Spouses who engaged in more work-related rumination reported less relationship satisfaction^22^ and worse marital interactions^23^ due to paying less attention to and being less psychologically available for one’s spouse. A similar relationship was found in the parent-child dyad, where parents who engaged in more work-related rumination engaged in worse interactions with their child through less psychological availability for their child^23^.

By continuing to think about work during leisure hours, engagement in work-related rumination extends strains from the workday into one’s home life^24^ and may facilitate crossover. In a daily diary study of paramedics and their spouses, King and DeLongis^18^ found that paramedics’ engagement in ruminative thought linked higher workday stress levels to more marital tension, a key indicator of poor dyadic functioning^25^. For dog-owning employees, engagement in work-related rumination could facilitate the strains of job stress crossing over into the owner-dog relationship. Ruminating about work may lead owners to pay less attention to their pets (as they did to their spouses^22^) and provide less care, resulting in an impaired pet-owner relationship and a more stressed pet. Ruminating employees may also emit emotional cues and even stress-related hormones^26^ that communicate the owner’s stress to the pet dog, which has translated to higher canine stress^12,13,27^.

Recently, scholars in organizational literature called out the importance yet neglect of understanding how pets, owned by the majority of Americans^9^, intersect with work life^28^. To our knowledge, the impact of owners’ job-related stress on their pets has not been explored. Thus, this study aims to shed light on a potential source of stress in pet dogs. Dogs may also be particularly vulnerable to a crossover effect due to being more socially aware of and in tune with humans than any other animal^11^. In this correlational study, we evaluate whether owners with more work-related stress have pet dogs who are more stressed. We also propose and evaluate work-related rumination as a mechanism by which an employee’s job stress may cross over to influence their dog. We measure pet dogs’ typical stress level in two ways, first as the owner’s perception of their dog’s stress level and second through owner observations of canine behaviors indicative of stress, as a less subjective measurement of stress.

### Hypothesis 1

Dog owners who experience more job stress will have a pet dog who is more stressed.

### Hypothesis 2

Dog owners with more job stress will have a pet dog who is more stressed due to higher engagement in work-related rumination.

## Method

All methods pertaining to this study involving human subjects were carried out in accordance with relevant guidelines and regulations of the Institutional Review Board (IRB) of Virginia Tech. Approval of all experimental protocols was issued by the Virginia Tech IRB (IRB #24-538). Data were collected during June and July of 2024. Employed dog owners were recruited through flyers, social media, and snowball sampling. On QuestionPro, an online survey management tool, participants provided informed consent and answered screening questions. Qualifying participants then took an online survey that asked about their dog and themself. They were instructed (and recurrently reminded) to select one dog to focus on for the duration of the survey. Participants’ free response answers were reviewed to screen for inauthentic respondents, and participants were screened out for not providing information on the focal variables (*n* = 8).

The final sample contained 85 employed pet dog owners (*N* = 85). Two-thirds of the owners were women (67%), and the average age was 38 years old (*SD* = 13.8). Most lived with other people (85%) and had only the one pet (59%). About a third of owners had children in the home (31%). Most owners typically worked about 40 h per week (*M* = 39.5, *SD* = 13.1), with about half of owners working from home at least occasionally (54%). Many participants were in a top management or supervisory position (38%), which aligns with a tendency of high earners to own a pet^29^. Participants were largely in either healthcare and social services (34%), professional, scientific, and technical services (20%), hospitality (12%), and trade (8%) work. The average age of pet was 7 years old (*SD* = 4.1), and the average time of ownership was 6 years (*SD* = 3.7). A quarter of dogs had been diagnosed with stress or anxiety issues, which aligns with previous rates found in dogs in the US^30^. More information about the dogs’ household and how they spend the day when their owner is working (e.g., alone) is given in Supplementary Table S1.

### Measurement tools

#### Pet dog stress

The dog’s typical level of stress was measured in two ways: via a visual analog scale (VAS), which in effect measured the owner’s perception of their pet’s level of stress, and via a behavior-based stress measure, which aimed to achieve an objective assessment of a pet’s level of stress.

##### Owner’s perception of pet dog stress

The Stress VAS (SVAS), used in both applied research and clinical contexts to evaluate one’s perceived level of stress^31^, instructs participants to “Indicate how stressed you feel” on a 100 mm ruler with endpoints “none” and “the most severe imaginable”^32^. Therapy dogs’ handlers have similarly reported their dog’s stress level using a single item scale, with agreement with dogs’ salivary cortisol levels^33^. In the present study, owners were asked “In general, how stressed is your pet?” and slid a bar along a horizontal line ranging from 0 to 100, with “0 is not at all; 100 is most severe imaginable,” to rate their dog’s stress level.

##### Behavioral indicators of pet dog stress

Given that owners are sometimes poor evaluators of their dog’s stress level^34^, owners were also asked to report how often their dog typically emits 11 stress-related behaviors. The aim of this assessment was to use pet behaviors that owners can identify even if they do not recognize them as stress indicators to gauge their dog’s stress level^35^ to achieve a more pet-led, unbiased measurement of stress.

Shown in Table 1, an 11-item list of behavioral stress indicators was derived from prior empirical work evaluating canine stress. From a multitude of stress-linked behaviors, 11 were selected as stress indicators for the current study based on multiple empirical studies indicating their association with stress^33,34,36–39^, literature suggesting that the behaviors occur in the presence of owners^33,34,36,40^, and evidence that owners recognize these behaviors^35^. Most behaviors were derived from studies of common stress indicators emitted by pet dogs in potentially stressful situations with their owners/handlers^33,34,36^, such as a vet visit^34^. Many of these behaviors were observed among dogs who had been subjected to conditions assumed to induce chronic stress^37,38^. Most of these indicators also appear in the Canine Behavioral Assessment and Research Questionnaire’s (C-BARQ^40^ “mild to moderate fear” level of the “fear and anxiety” section, which describes behaviors frequently observed in response to typical canine stressors (e.g., thunderstorms). More detail on how the 11 behaviors were selected is provided in the Supplementary Information Appendix.

**Table 1:** Behavioral indicators of stress for pet dogs and supporting literature.

Behavioral indicator of stress	Examples of literature support
Excessive nose or lip licking	Clark et al. (2020)^33^, Mariti et al. (2015)^34^, Mariti et al. (2012)^35^, Beerda et al. (1999)^37^
Wet dog shake	Clark et al. (2020)^33^, Mariti et al. (2015)^34^, Beerda et al. (1999)^37^, Beerda et al. (2000)^38^, Hsu and Serpell (2003)^40^
Yawning	Clark et al. (2020)^33^, Mariti et al. (2015)^34^, Mariti et al. (2012)^35^, Beerda et al. (2000)^38^
Leaning into people	Clark et al. (2020)^33^, Dreschel and Granger (2005)^36^, Beerda et al. (2000)^38^
Crying, whimpering, or whining	Mariti et al. (2015)^34^, Mariti et al. (2012)^35^, Beerda et al. (1999)^37^, Dreschel and Granger (2005)^36^, Hsu and Serpell (2003)^40^
Avoiding eye contact	Mariti et al. (2012)^35^, Hsu and Serpell (2003)^40^
Tail lowered or tucked between the legs	Mariti et al. (2015)^34^, Beerda et al. (1999)^37^, Beerda et al. (2000)^38^, Hsu and Serpell (2003)^40^
Panting	Clark et al. (2020)^33^, Mariti et al. (2012)^35^, Dreschel and Granger (2005)^36^, Beerda et al. (1999)^37^
Poor appetite	Mariti et al. (2012)^35^, Luño et al. (2018)^39^, Hsu and Serpell (2003)^40^
Inappropriate urinating or defecating	Mariti et al. (2012)^35^, Beerda et al. (1999)^37^, Dreschel and Granger (2005)^36^, Beerda et al. (2000)^38^
Excessive walking or pacing	Mariti et al. (2015)^34^, Mariti et al. (2012)^35^, Dreschel and Granger (2005)^36^, Beerda et al. (1999)^37^, Beerda et al. (2000)^38^, Hsu and Serpell (2003)^40^

Synthesizing this literature, we composed the list of 11 stress indicators shown in Table 1 as a behavioral measure of dog stress. Dog-owning participants were asked “How often does your dog emit the following behaviors” on a 5-point Likert-type scale ranging from “Never” to “All the time” (Cronbach’s α = 0.632). The final behavioral score was the average of the 11 items, thus scores range from low stress at 1 to high stress at 5.

### Job stress

The owner’s level of job stress was evaluated using the Subjective Job Stress Scale (SJSS^41)^. The SJSS contains four items on perceived level of stress arising from one’s job measured on a 5-point Likert-type scale ranging from “Strongly disagree” to “Strongly agree.” An example item is “Very few stressful things happen to me at work” (Cronbach’s α = 0.858).

### Work-related rumination

Perseverative thoughts about work during leisure time were measured using the Work-Related Rumination Scale^42^. Given its relevance both to emotional contagion and stress, participants specifically completed the five-item affective rumination subscale, which focuses on one’s negatively emotional thoughts about work experienced during non-work time^24^. An example item is “Are you irritated by work issues?” Items were measured on a 5-point Likert-type scale ranging from “Strongly disagree” to “Strongly agree” (Cronbach’s α = 0.880).

### Home stress

To isolate stress due to one’s job, we statistically controlled for stress arising from one’s home life using a scale developed to measure home-related stress in Fan et al.^43^. To mirror the scale of subjective job stress used in the current study, we used the self-perceived stress at home subscale. This subscale contains four items, such as “I feel stress at home,” measured on a 5-point Likert-type scale ranging from “Strongly disagree” to “Strongly agree” (Cronbach’s α = 0.749).

### Analytic approach

All composite variables were computed by taking the mean score of items. To evaluate Hypothesis 1 that tested the direct effect of job stress on pet stress, we used general linear modeling (GLM). The focal independent variable of job stress and the control variable of home stress were entered as predictors of a single criterion. The two criterion variables, perception- and behavior-based stress, were entered into the model separately. To evaluate Hypothesis 2 that tested the indirect effect of job stress on pet stress through work-related rumination, we performed path analyses with bootstrapping. Job stress and home stress were input as predictor variables, with job stress serving as the focal independent variable and home stress serving as a control variable. Work-related rumination was input into the models as a mediator variable, and the dependent variable was owner-perceived pet stress and the behavioral measure of stress, modeled separately. These analyses were performed with 10,000 bootstrapped samples. All analyses were computed in Mplus Version 8.4^44^.

## Results

One outlier on the behavioral stress measure was removed from all analyses due to its z-score exceeding the 3.29 threshold recommended by Tabachnick and Fidell^45^. Correlations, means, and standard deviations are shown in Table 2. The skewness and kurtosis for each variable remained within a range of ± 1.5, suggesting normality in the variable distributions^45^. Perceived dog stress was only moderately correlated with behavioral dog stress (*r* = 0.335, *p* = 0.002). Thus, the perceptual and behavioral measures of dog stress may comprise separate constructs. On a scale of 0–100, participants reported perceiving an average stress level of *M* = 28.929 (*SD* = 22.488, range: 0–88) in their dogs, aligning with a low-to-moderate level of stress. Behaviorally speaking, participants observed that, on average, their pets emitted stress-related behaviors “Rarely” (*M* = 2.061, *SD* = 0.433, range: 1.18–3.00).

**Table 2:** Descriptive statistics and inter-correlations of study variables.

	M	SD	1	2	3	4	5
Focal variables							
1. Job stress	3.050	1.095	–				
2. Work-related rumination	2.511	0.889	0.600**	–			
3. Perceived dog stress	28.929	22.354	− 0.046	0.014	–		
4. Behavioral dog stress	2.061	0.433	0.278*	0.404**	0.335**	–	
Control variable							
5. Home stress	1.635	0.706	0.051	0.114	0.257*	0.254*	–

*p < 0.05, **p < 0.01.

**Fig. 1:**
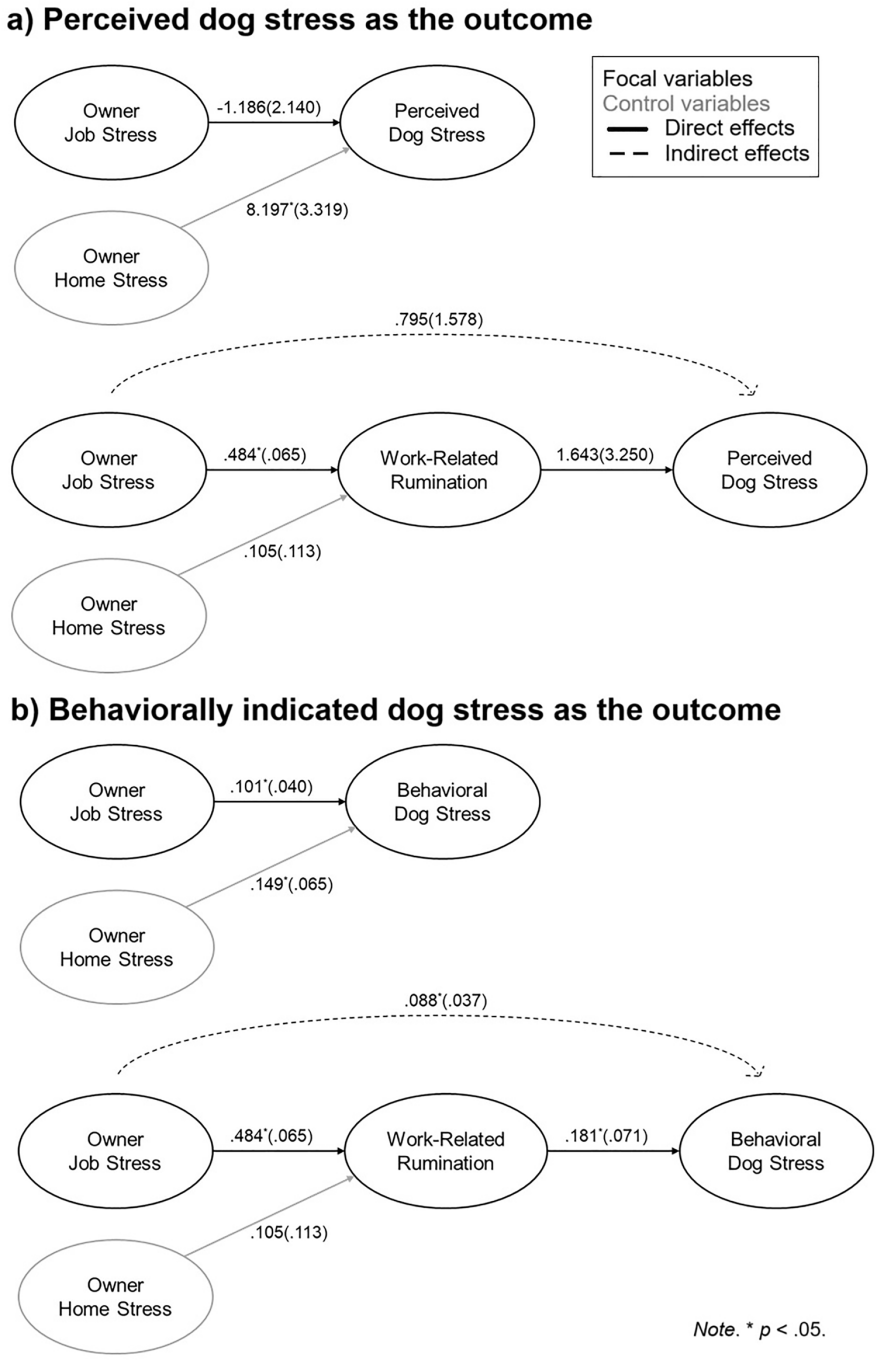
Estimated models: Evaluating the link from an owner’s job stress to their dog’s stress directly and indirectly through work-related rumination. Figure displays the four models estimated in this study. Panel (**a**) shows the direct and indirect effects models with perceived dog stress as the outcome, and panel (**b**) shows the direct and indirect effects models with behaviorally indicated dog stress as the outcome. Focal variables are marked in black, while the control variable is marked in gray. Direct effects are shown with solid lines, while indirect effects are illustrated with dashed lines. Path coefficients (marked with statistical significance) and standard errors are provided.

**Fig. 2:**
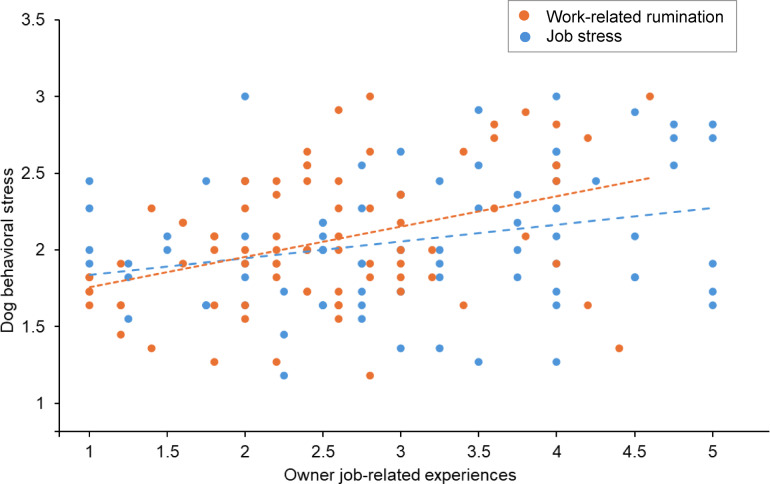
Dogs’ behaviorally indicated stress as predicted by their owner’s job stress and work-related rumination. Figure shows two overlaid scatterplots, each depicting a relationship between an owner’s job-related experience and their dog’s behaviorally indicated stress. One set of data points illustrates the relationship between owners’ job stress and their dog’s behaviorally indicated stress (in blue), and the other shows the relationship between owners’ work-related rumination and their dog’s behaviorally indicated stress (in orange). A respective fit line goes through each set of data points.

Estimated models are shown in Fig. 1. As shown in Supplementary Table S2, we found support for a direct association between job stress and behaviorally indicated dog stress (*γ* = 0.101, *SE* = 0.040, *z* = 2.534, *p* = 0.011) but not for perceived dog stress as the criterion (*γ* = − 1.186, *SE* = 2.140, *z* = − 0.554, *p* = 0.579). The simple scatter plot for job stress and behavioral dog stress is shown in Fig. 2. Although input into the model as a control variable, we note the link detected between an owner’s home-related stress and their dog’s stress, both in terms of perceived dog stress (*γ* = 8.197, *SE* = 3.319, *z* = 2.470, *p* = 0.014) and behavioral dog stress (*γ* = 0.149, *SE* = 0.065, *z* = 2.281, *p* = 0.023).

As shown in Supplementary Table S3, we found support for an indirect association between job stress and behavioral dog stress through work-related rumination (*γ* = 0.088, *SE* = 0.037, *z* = 2.353, *p* = 0.019). The simple scatter plot for work-related rumination and behavioral dog stress is shown in Fig. 2. With the inclusion of work-related rumination in the model, no direct effect between job stress and dog stress remained (*γ* = 0.022, *SE* = 0.053, *z* = 0.415, *p* = 0.678), suggesting full mediation^46^. However, no indirect effect was found between job stress and perceived dog stress via work-related rumination (*γ* = 0.795, *SE* = 1.578, *z* = 0.504, *p* = 0.615).

## Discussion

This study uncovers another victim of an employee’s job stress: the employee’s pet dog. In addition to the physical and mental well-being impairments that relate to high job stress^2^, job stress can impact loved ones through crossover^8^. In partial support of our hypotheses, we found that dogs owned by more job-stressed owners displayed more behavioral indicators of stress (Hypothesis 1), specifically by way of their owner’s work-related rumination (Hypothesis 2). To our knowledge, this is the first study to support the phenomenon of crossover in an employee-pet relationship. Given that 97% of Americans view their house pets as family members^9^, protecting one’s pet dog may serve as a powerful motivator to improve one’s level of work stress and resist engaging in ruminative thoughts about work.

The means by which job stress associated with dog stress was work-related rumination, or the engagement in continued thoughts about work during off-job hours. Engagement in ruminative thoughts about work may make owners less mindful and supportive of their dogs’ needs, as was shown in human dyads^6,22,23^, which could result in a dog who is more stressed. This reasoning aligns with the indirect transmission of crossover proposed by Westman^8^, wherein crossover occurs due to side effects of the stress experienced by the employee.

Work-related rumination could also facilitate the direct transmission of crossover offered by Westman^8^, wherein dogs may “catch” their owner’s job stress more when the owner is ruminating over work more. Owners may show more behavioral signs of stress when mentally engaging with work stressors. Like dogs, humans emit certain behavioral patterns when experiencing negative emotions such as using particular facial expressions^47^, bodily movements and postures^48^, and vocalizations^49^. Experimental manipulations of each of these modalities have demonstrated that dogs can interpret and react to such emotional cues from humans^15,50–52^. Dogs also appear to engage in social referencing, in which they look to their owner to determine how they should interpret a situation^53^. Therefore, as an owner spends more time muddling through negatively emotional thoughts, they likely send more negatively emotional signals that their dogs may perceive to their own detriment.

In addition to behavioral changes, dogs may even perceive physiological changes induced by work-related rumination. A study of dogs’ responses to the smells left by stressed versus relaxed human strangers indicated that dogs behave differently in response to, and thus differentially perceive, stressed versus non-stressed human odors^54^. Prior research has demonstrated that rumination can induce changes in cortisol^26^, and perhaps dogs perceive this change in their owners and “catch” the job-related stress.

Turning to measures, we found an interesting discrepancy between owner-perceived and behaviorally indicated levels of canine stress. These two measurements of stress were only moderately correlated (*r* = 0.335, *p* < 0.01) despite intending to measure the same construct. One interpretation of these results is that dog owners are not acutely tuned into their pets’ stress levels. A study by Mariti and colleagues^35^ on how well dog owners know canine behavioral signs of stress suggests that dog owners are lacking in knowledge, with the majority of dog owners only successfully identifying two of 19 stress-related behaviors. Dogs’ label of “man’s best friend” may result in human stereotypes and biases towards their dog’s behavior, such as an owner feeling that they understand their dog even if the reality is not so. Dogs have been shown to be vulnerable to anthropomorphization, which can bias owners’ attitudes toward their dogs^55^. Specifically, owners who viewed their dogs like children believed their dogs to be more empathetic and responsive to their own behavior. These biases could make dog owners particularly likely to overlook behavioral signs of stress in favor of their own projections of their pet’s stress level.

As a potential limitation, our behavioral measure of canine stress comprised 11 indicators based on prior empirical work, but more canine stress indicators exist. Other indicators may be emitted more consistently, noticed by owners more reliably, or more representative of stress in chronic (the focal setting of the current study) versus acute (e.g., a veterinary waiting room) settings and thus would pose better construct validity for the current context. We do note that dogs experiencing chronic stress may emit behaviors reflective of acute stress in response to the presence of a familiar human^38^, supporting our partial reliance on indicators derived from acutely stressful events^34,36^. More work on the nature of chronic stress is needed^56^ and could be enhanced by the inclusion of physiological measures.

Measurement error could also stem from inconsistencies due to reliance on owner-report and lack of a specific timeframe posed in the behavioral measure. Although the question asked “How often does your dog emit the following behaviors,” how and when owners observed their dog’s behaviors could vary, and differences in how much time owners spend with their dogs could impact how often they see stress behaviors. Additionally, the Cronbach’s α for the current sample was relatively low at 0.632; although, as this scale is formative rather than reflective, we do not believe that this value informs the measure’s construct validity. Finally, the sample used was a convenience sample not representative of all pet dogs. A larger, more systematically sampled study would improve our understanding of the relationship between owners’ and pet dogs’ stress.

Overall, this study adds to our understanding of how pets intersect with work life and pinpoints an area of concern for canine welfare. Employees may wish to avoid engaging in ruminative thoughts about work when at home by practicing mindfulness^57^, performing transition rituals to keep work and home separate^58^, or spending time actively engaging with their dogs to potentially combat crossover to their dogs. Future research could test whether such active engagement correlates with benefits for both the owner (i.e., in terms of reduced rumination) and their dog (especially as prior empirical evidence suggests that physical activity can reduce stress in dogs^17^).

This study also adds to the growing body of support that dog stress is correlated with their owners’ stress, which supports the notion of heterospecific emotional contagion between dogs and their owners. We observed this phenomenon with regard to a chronic and typical form of stress, rather than an acute and event-based stress. Furthermore, this study has ecological relevance by pertaining to the home context as opposed to a lab space, which is an issue found in many studies of heterospecific emotional contagion^13^. Examining factors related to the home context, such as presence of other pets and children, as well as to the work context, especially flexibility to work from home, could reveal additional paths for weakening a link between an owner’s job stress and their pet’s stress.

## Conclusion

This study united companion animal welfare with organizational research to explore how an employee’s job stress may relate to their pet dog’s stress. A crossover effect has been observed between employees and their spouses and children, wherein the stress experienced by an employee due to their job is felt by their loved ones^3,6,8^. This phenomenon, to our knowledge, had not yet been studied between employees and their pets despite the close bond that owners and pets share. Furthermore, support exists for emotional contagion between owners and pets^13^, suggesting that pets can absorb their owner’s feelings and thereby may be vulnerable to their owner’s job stress.

This study provides support for a crossover effect between an employee and their pet dog, a valued family member^9^ that has been neglected by organizational literature^28^. We found that employees with more job stress owned dogs who themselves showed more behavioral signs of stress. This crossover effect was explained by an owner’s tendency to engage in work-related rumination, or continued thoughts about work after work. Prior work on rumination suggests that this tendency may remove the owner’s attention from their pet^22^, leading to less care, or may induce physiological and behavioral changes that communicate the owner’s negative feelings to their dog^26^. We encourage dog owners to combat work-related rumination through targeted interventions like mindfulness practice and being intentional in using leisure time for leisure.

## Electronic supplementary material

Below is the link to the electronic supplementary material.

Supplementary Material 1

## Data Availability

Data are provided in Supplementary Table S4.
