# Supplementary material for: Dog owners’ job stress crosses over to their pet dogs via work-related rumination
Source: Sci Rep. 2025 May 15;15:16887. doi: 10.1038/s41598-025-01131-x (PMC12081690; doi:10.1038/s41598-025-01131-x)
Supplement: Supplementary file 1 — Supplementary Material 1 [file 41598_2025_1131_MOESM1_ESM.pdf]

Dog owners’ job stress crosses over to their pet dogs via work-related rumination

Tanya Mitropoulos & Allison Andrukonis

Supplementary Information

Appendix
Behavioral Indicators of Pet Dog Stress

Owners were asked to report how often their pets typically emit behaviors that are indicative of stress. Prior research has shown that owners are often poor evaluators of their dog's stress level [34]. So, we supplemented the owner-perceived stress measurement above with a more pet-led measurement of stress. The aim of this assessment was to use pet behaviors that owners can identify even if they do not recognize them as stress indicators to gauge their pet's stress level [35].

An 11-item list of behavioral stress indicators was derived from prior empirical work evaluating canine stress. A multitude of behaviors have been described as indicating stress. We narrowed down possible behaviors to 11 by looking for behaviors that were listed consistently (i.e., in multiple empirical works), are not exclusively linked to trauma responses (as the stress evaluated in this study is chronic [i.e., typical] in nature and not necessarily severe), and have empirical evidence suggesting that owners recognize these behaviors. In line with the goal of choosing indicators that owners notice, we also targeted empirical studies examining canine stress-linked behaviors in the company of a human companion.

In an empirical study of dog stress, Clark et al. [33] followed nine therapy dog-handler teams on a hospital visit. During these visits, the research team noted the most commonly observed stress-related dog behaviors. These behaviors included "panting, lip licks, yawning, leaning into people, turning away from a stimulus, and 'wet dog' shake" (p. 6). Similarly, in Mariti et al. [34], two researchers coded 3-minute video recordings of 45 dogs in a veterinary clinic waiting room to assess which stress indicators were most commonly shown by pet dogs. The researchers marked the proportion of dogs that emitted each of 19 pre-selected common stress indicators. As in Clark et al. [33], panting and yawning were two of the most common stress-related behaviors emitted, with over half of dogs panting during the 3-minute segment and over a third yawning. By far the most common stress indicator in Mariti et al. [34] was nose licking, which mirrors the activity of lip licking identified in Clark et al. [33]. Lowered ears and crying were also frequently seen in Mariti et al. [34], with nearly half of dogs showing these two signs of stress. Interestingly, leaning into people and turning away from a stimulus, common signs seen in Clark et al. [33], were not among the 19 behaviors coded for in the dogs at the vet offices.

We also considered in developing this measure which behaviors owners recognize as indicating stress. Mariti et al. [35] asked 1190 dog owners to mark which of 19 stress-related behaviors they recognized as indicating stress. Dog owners most consistently recognized trembling, crying/whining, aggressiveness, excessive barking, and panting as stress indicators. Between a third and a fifth of the sample recognized high activity, low activity, low appetite, hypersalivation, and inappropriate urination and defecation as stress indicators.

In a home setting, Dreschel and Granger [36] examined how pet dogs react to thunderstorms when with their owner. These researchers observed the dogs to show many of the stress-related signs that owners report recognizing [35]. In particular, their sample of 19 dogs commonly paced, cried, panted, and one dog defecated following exposure to a simulated thunderstorm.

Beerda and colleagues performed two studies that focused on canine behavioral responses to chronic stress [37, 38]. One sample (72 dogs) had lived for years in four types of housing conditions assumed to induce different levels of chronic stress [38]. The dogs who had lived in the conditions assumed to be the most stressful displayed high levels of pacing and urinating, as well as nosing and paw-lifting, when not around humans. In the presence of humans, the authors noted that this group of dogs emitted behaviors typically associated with *acute* stress, such as the

wet dog shake, yawning, and certain postures like having a lowered tail. Another sample of dogs (15 beagles) was subjected to social and spatial restriction for 6 weeks, after which behavioral observations were recorded during outdoor group and indoor solitary contexts [37]. In this study, interaction with humans was minimized. The researchers reported many of the same behaviors as in the aforementioned studies, such as crying, lowered tail, pacing, increased elimination (i.e., urinating and defecating), and wet dog shake. The authors also noted the presence of more extreme behaviors like coprophagy, aggression, and stereotypies that suggest an intensive level of stress likely stemming from the dogs' severe living conditions.

The Canine Behavioral Assessment & Research Questionnaire (C-BARQ [40]) is a very widely used questionnaire used not only by animal professionals but also by pet owners to evaluate dogs' temperament and behavior. While the questionnaire does not directly measure stress level, it does contain a "Fear and anxiety" section that describes behaviors frequently observed in response to typical canine stressors, such as being approached by an unfamiliar person, heavy traffic, thunderstorms, and having nails clipped. The C-BARQ lists behaviors representative of "mild to moderate fear," which we consulted for this study rather than those pertaining to "extreme fear" due to relevance to the current context. Those mild-to-moderate behaviors largely echoed those identified in empirical work, including avoiding eye contact, tucking the tail between the legs, and whimpering. The C-BARQ's separation-related behavior section also relates to signs of anxiety in dogs, but specifically with respect to abnormal behaviors shown when the owner is gone or about to leave. Many of these behaviors are forms of vocalizations and also include restlessness, pacing, and loss of appetite.

Synthesizing this literature, we composed the following list of 11 stress indicators as a behavioral measure of dog stress. We asked dog-owning participants "How often does your dog emit the following behaviors" on a 5-point Likert-type scale ranging from "Never" to "All the time."

1. Excessive nose or lip licking
2. Wet dog shake
3. Yawning
4. Leaning into people
5. Crying, whimpering, or whining
6. Avoiding eye contact
7. Tail lowered or tucked between the legs
8. Panting
9. Poor appetite
10. Inappropriate urinating or defecating
11. Excessive walking or pacing

Supplementary Table S1

Characteristics of Dog's Home and Day Spent During Owner's Work

	Proportion of Sample
**Household characteristics**	
Other pet(s)	41%
Other human(s)	85%
Child(ren) in the home	31%
Child(ren) under age 5	20%
Yard for playing outside	71%
**Characteristics of dog's workday***	
Alone (no pets, no people)	16%
With other pets, but not people	15%
Some days with people, some days without (typically due to hybrid work arrangements)	27%
With people everyday	31%
At doggy daycare at least occasionally	7%
In the office with owner	2%

Note. * Data are from free responses to the question "During work hours, typically where is your pet and who is your pet with (including other pets)?"

Supplementary Table S2

Direct Effect of Job Stress on Dog Stress (Hypothesis 1)

	Perceived Dog Stress[†]			Behavioral Dog Stress[‡]		
	Est.	*SE*	*z*	Est.	*SE*	*z*
Intercept	19.170*	8.593	2.231	1.510**	.161	9.392
Statistical Control						
Home stress	8.197*	3.319	2.470	.149*	.065	2.281
Focal Variable						
Job stress	-1.186	2.140	-.554	.101*	.040	2.534
Residual variance	464.876**	71.732	6.481	.161**	.025	6.481
Pseudo-R^2	.070	.054	1.300	.131	.069	1.908

Note. * $p < .05$. ** $p < .01$.

† Perceived dog stress was measured on a scale of 0-100, with higher values representing higher stress.

‡ The behaviorally indicated measure of dog stress was measured on a scale of 1-5, with higher values representing higher stress.

Supplementary Table S3

Indirect Effect of Job Stress on Dog Stress through Work-Related Rumination (Hypothesis 2)

	Work-Related Rumination			Perceived Dog Stress†			Behavioral Dog Stress‡		
	Est.	*SE*	*z*	Est.	*SE*	*z*	Est.	*SE*	*z*
Intercept	.864*	.257	3.367	30.074*	8.719	3.449	1.535**	.134	11.425
<u>Statistical Control</u>									
Home stress	.105	.113	.928						
<u>Focal Variable</u>									
Job stress	.484*	.065	7.404	-1.735	2.681	-.647	.022	.053	.415
Work-related rumination				1.643	3.250	.506	.181*	.071	2.542
Simple indirect effect				.795	1.578	.504	.088*	.037	2.353
Residual variance	.500**	.064	7.771	497.315**	64.815	7.673	.155**	.022	7.089
Pseudo-R^2	.367**	.070	5.266	.005	.026	.184	.168*	.083	2.012

Note. * $p < .05$. ** $p < .01$.

† Perceived dog stress was measured on a scale of 0-100, with higher values representing higher stress.

‡ The behavioral measure of dog stress was measured on a scale of 1-5, with higher values representing higher stress.

Supplementary Table S4

Supplementary Dataset

Perceived Dog Stress	Behavioral Dog Stress	Employee Job Stress	Work-Related Rumination	Home Stress
20	2.09	1.5	2.2	1
0	1.27	4	2.2	1
40	2.45	1	2.2	3
49	1.55	2.75	2.6	2.75
38	2.64	4	2.8	2.75
12	2.36	3	3	1.5
24	2.27	2.75	1.4	2
0	1.36	3	1.4	1.5
20	2	3.25	3.2	1.25
21	1.91	2	2.2	1.75
66	3	2	2.8	1.75
0	1.64	1.75	1.2	1.25
50	3	4	4.6	2
28	2.55	3.5	4	1.25
75		2.25	1	3.75
33	1.64	2.75	2	1
20	1.36	3.25	4.4	2
26	2.36	3.75	2.2	3.25
70	1.91	1.25	2.2	1.25
20	2.27	3.5	2.8	1
0	2.09	2.5	1.8	1
28	1.82	3.25	3.2	2.25
55	1.64	2	1.2	1.75
75	2	3	2.2	1
15	1.82	4.5	3	1
1	1.73	2.25	2.6	1.75

0	1.91	1	1.2	1.25
62	1.82	1.25	1	1
65	2.82	5	4	1.5
15	1.64	1.75	2.6	1.25
20	1.82	4.5	2.2	1
66	2	3	3	1.25
12	1.73	5	3	1
10	1.91	4	1.6	1
50	2.45	1.75	2	2.25
65	2.55	4	2.4	2.25
50	1.64	2.5	1	1
52	1.91	5	4	1.75
15	2.27	4	3.6	1.25
0	2.09	4	1.8	1
0	1.55	1.25	2	1
20	2	2.5	2.4	2.25
50	1.73	3	1	1
15	2	3.75	2.4	1.25
10	1.64	2.5	1.8	3.75
25	1.73	2.75	1	1.5
50	2.55	2.75	2.4	2.25
5	1.82	3.75	2.8	2.25
66	2.55	4.75	4	2.25
61	2.64	3	2.4	1.75
60	1.73	4	2.4	1
5	1.91	2.75	2.8	1
30	1.64	5	4.2	1
51	2.45	3.25	2.4	3
23	2.73	5	3.6	2.5
20	2	2.5	2	1
88	2.45	2	2	1
16	2.27	4	2.6	1

1	1.45	2.25	1.2	1
25	2.45	4.25	4	1.25
1	2.9	4.5	3.8	1.5
20	2	1	2.6	1.25
30	2.36	3	3	3.25
50	1.64	1.75	2.6	1.75
30	1.82	2	1	1
20	2.27	1	2	1
10	1.64	4	2.6	1.75
15	2.73	4.75	4.2	1.75
20	1.91	2.75	3	1
30	2.18	2.5	1.6	1.25
20	2.09	4.5	3.8	2
11	2.18	2.5	1.6	1
30	2.82	4.75	3.6	1.5
	1.27	3.5	1.8	2
12	2.45	4	2.6	1
20	2	2.5	2.4	1
24	2	1.5	1.8	1
12	1.18	2.25	2.8	1.25
20	1.64	2.5	3.4	1.75
41	2.18	3.75	3	2.5
72	2.91	3.5	2.6	2.75
0	1.91	3.25	2	1.25
25	2.09	2	2.6	2.5
4	2.64	4	3.4	1.75
24	1.73	3	2.4	2.75
